# Genetic predisposition for vitamin D deficiency is not associated with adverse outcome of very low birth weight infants: A cohort study from the German Neonatal Network

**DOI:** 10.1371/journal.pone.0230426

**Published:** 2020-03-31

**Authors:** Clara Mannhardt, Tanja K. Rausch, Mats Ingmar Fortmann, Isabelle Swoboda, Alexander Humberg, Juliane Spiegler, Wolfgang Göpel

**Affiliations:** 1 Department of Pediatrics, University of Lübeck, Lübeck, Germany; 2 Institute of Medical Biometrics and Statistics, University of Lübeck, Lübeck, Germany; Centre Hospitalier Universitaire Vaudois, FRANCE

## Abstract

**Objective:**

Postnatal vitamin D supplementation is standard of care in neonates and preterm infants. Despite routine supplementation of vitamin D, a wide range of complications related to vitamin D deficiency has been described in the literature. Since standard vitamin D supplementation might be not sufficient in preterm infants with a genetic predisposition for vitamin D deficiency, we investigated the outcome of preterm infants with regard to their genetic estimated vitamin D levels.

**Methods:**

Preterm infants with a birth weight below 1500 grams were included in the German Neonatal Network at the time of their birth and tested at the age of five. The vitamin D level was genetically calculated based on three single nucleotide polymorphisms (SNPs: rs12794714, rs7944926 and rs2282679) which alter vitamin D synthesis pathways. Specific alleles of these polymorphisms are validated markers for low plasma vitamin D levels. Outcome data were based on baseline data at the time of birth, typical complications of prematurity, body measurements at the age of five and occurrence of bone fractures. T-test and Fisher’s exact test were used for statistical comparison.

**Results:**

According to their genetic predisposition, 1,924 preterm infants were divided into groups of low (gsVitD < 20. Percentile), intermediate and high vitamin D level estimates. Low genetic vitamin D level estimates could not be shown to be associated with any adverse outcome measures examined. The analyses covered data on aforementioned determinants.

**Conclusion:**

Low genetic vitamin D level estimates could not be shown to be associated with previously described adverse outcome in preterm infants.

## Introduction

Vitamin D deficiency might negatively impact prematurity due to its wide range of effects [1], including obesity, diabetes mellitus, allergies, cancer, arterial hypertension and increased risk for neurological complications [2–8]. In addition, vitamin D has anti-proliferative, anti-inflammatory and organ-protective effects [2]. The best-known detrimental effect of a vitamin D deficiency is an imbalance in the bone homoeostasis, which commonly leads to the development of rickets in children [9].

Vitamin D deficiency is a worldwide and currently discussed health problem, which affects more than a billion children and adults. Holick defines a vitamin D deficiency as 25(OH)D levels below 20ng/ml and levels between 21 and 29ng/ml as insufficiency. Levels above 30ng/ml are consequently classified as sufficiency [9].

The effect of vitamin D is based on the increase of the concentrations of calcium and phosphate in the kidneys, the intestines and the bones and enhances the turnover of the bone matrix and the mobilization of calcium and phosphate from the bone. This contributes to a healthy bone growth and a balanced bone homoeostasis [2]. Since in adults the main growth of the bones is finished and the epiphyseal plates are closed, a vitamin D deficiency results in osteomalacia. This leads to bone pain and an increased risk of fractures [10]. Calcium independent symptoms of a vitamin D deficiency exist and can be explained by a ubiquitous occurrence of the vitamin D receptor [1].

In 2010, 14.9 million children were born as preterm infants (birth < 37+0 week of pregnancy), which accounts for 11.1% of all births worldwide [11]. Prematurity is associated with a variety of short-term complications: respiratory (bronchopulmonary dysplasia (BPD)), neurological (intraventricular hemorrhage (IVH), periventricular leukomalacia (PVL)) abdominal (necrotizing enterocolitis (NEC)) complications, retinopathy of prematurity as well as sepsis and rickets of prematurity. These complications are common and determine the prognosis of preterm infants [12]. Reduced cerebellar volume, reduced intelligence quotient, cognitive impairments, behavioral problems, higher prevalence of arterial hypertension, childhood asthma and type 2 diabetes are long term complications of prematurity [13].

We hypothesize that, considering the negative effects of vitamin D deficiency, a genetic predisposition to low vitamin D levels is detrimental to the outcome of premature infants. This hypothesis is tested by using a genetic score for vitamin D levels with a special focus on the appearance of typical complications of prematurity and fractures at the age of five years.

## Methods

### Study design

Infants were included in the German Neonatal Network at the time of their birth and followed up at the age of five years. This study was approved by the ethics committee of the University of Lübeck, Germany (08–022) and at each participating center. Written informed parental consent was given for the research and publication of the results of each infant included in the study. The German Neonatal Network is a large-scale observational study with the aim to identify possible influencing factors for the outcome of preterm infants. The inclusion criteria were a birth weight below 1500g, a gestational age below 37 weeks + 0 days of pregnancy and a birth in one of the participating hospitals. During the stay in the hospital, the deoxyribonucleic acid (DNA) of the neonate was collected (umbilical cord tissue and buccal-swab). Clinical data were collected by standardized questionnaires and regular onsite monitoring of participating centres. All data were collected in a central database at the University of Lübeck.

At the age of five, about 60% of the infants cohort were randomly selected for follow-up. We used standardized test, to assess their growth, neurocognitive development, fine and gross motor skills, hearing and visual ability, lung function and intelligence quotient.

We excluded preterm infants with unsuccessful genotyping of three polymorphisms affecting vitamin D levels (see below) or incomplete follow-up data at five years. The most common reason for missing follow-up data was the age of the child, since all children with follow-up were born between 2009 and 2013, but genetic testing was done in children born between 2009 and 2015.

### SNP selection and DNA isolation

A number of SNPs with validated effects on vitamin D levels are described in the literature. For the purpose of this study we genotyped rs12794714, rs7944926 and rs2282679. These SNPs have well-establishes associations with a reduction of the 25(OH)D concentration: a single “A”-allele of rs12794714 reduces 25(OH)D by 3.0 nmol/l, an “A”-allele of rs7944926 by 2.0 nmol/l and the “C”-allele of rs2282679 by 2.5 nmol/l [6,7,14,15]. These SNPs encode enzymes which are important for the vitamin D synthesis pathway. rs12794714 is located on chromosome 11 in the CYP2R1-gene encoding the vitamin D 25-hydroxylase. The SNP rs7944926 is located on chromosome 11 as well, close to the DHCR7-gene encoding7-Dehydrocholesterol reductase. The SNP rs2282679 is on chromosome 4 in the GC (Group-Specific-Component)-gene, encoding the vitamin D binding protein [16]. To avoid contamination with maternal DNA, infant DNA from the umbilical cord tissue was isolated by using the Gentra^®^ Puregene^®^ Tissue Kit, while the QIAamp^®^ 96 DNA Kit (QIAGEN, Hilden) was used for the DNA isolation from buccal-swabs. SNPs were amplified by polymerase chain reaction and allelic discrimination was conducted with a sequence detecting system-software using the TaqMan^®^ 7900 HT platform (Applied Biosystems). Context sequences for primers were rs12794714: TTCTCATGTAGACATGGGGAAGCTC[A/G]GATGAGGCTGCCAGGGAATAGATGT; rs7944926:ATTTGCTCAGAGCAAATCTAGTTG[A/G]ACTGAAGAAGGCTTGGCCAAAACTA and rs2282679:AAAGCTAACAATAAAAAATACCTGGC[T/G]TGTGAGATAATTAAGAGACAGAGATTTGC. We used a two-step PCR protocol with 15 seconds at 92° Celsius and 60 seconds at 60° Celsius (40 cycles).

### Genetic score for vitamin D levels

A genetic score for vitamin D (gsVitD) levels was calculated by *gsVitD = (n*_1_* -3) + (n_*2*_ * -2) + (n_*3*_ * -2.*5)*, where the number of A-alleles of rs12794714 (0, 1 or 2) was entered as n_1_, the number of A-alleles of rs7944926 as n_2_ and the number of C-alleles of rs2282679 as n_3_. Thresholds for the 20^th^ and 80^th^ percentile of gsVitD were -7.6 and -2.9 nmol/l.

### Outcome variables

Infants with a birth weight or length below the 10^th^ percentile were defined as small-for-gestational-age (SGA) infants [17]. The intraventricular haemorrhage (IVH) is divided into four degrees of severity and ranges from bleeding in the germinal matrix to haemorrhagic infarction [18]. Periventricular leukomalacia (PVL) is caused by necrosis near the ventricles of the brain [19]. IVH and PVL was assessed by routine ultrasound of the brain. Bronchopulmonary dysplasia (BPD) was defined as the need of oxygen or respiratory support at 36+0 weeks of pregnancy [20]. For this work a blood culture proven bacteremia plus clinical signs was defined as sepsis and necrotizing enterocolitis was defined as necrosis of the intestine requiring surgery. Retinopathy of prematurity (ROP) was defined as any ROP with invasive treatment (laser- or cryotherapy or intraocular injection of VEGF-antibodies).

### Statistics

The statistical analysis was performed with SPSS^®^, version 22.0. We compared infants with gsVitD < 20^th^ percentile to infants with gsVitD > 80^th^ percentile by using t-test, Fisher’s exact test and the Bonferroni correction. The type I error level was set to 0.05.

## Results

**Fig 1:**
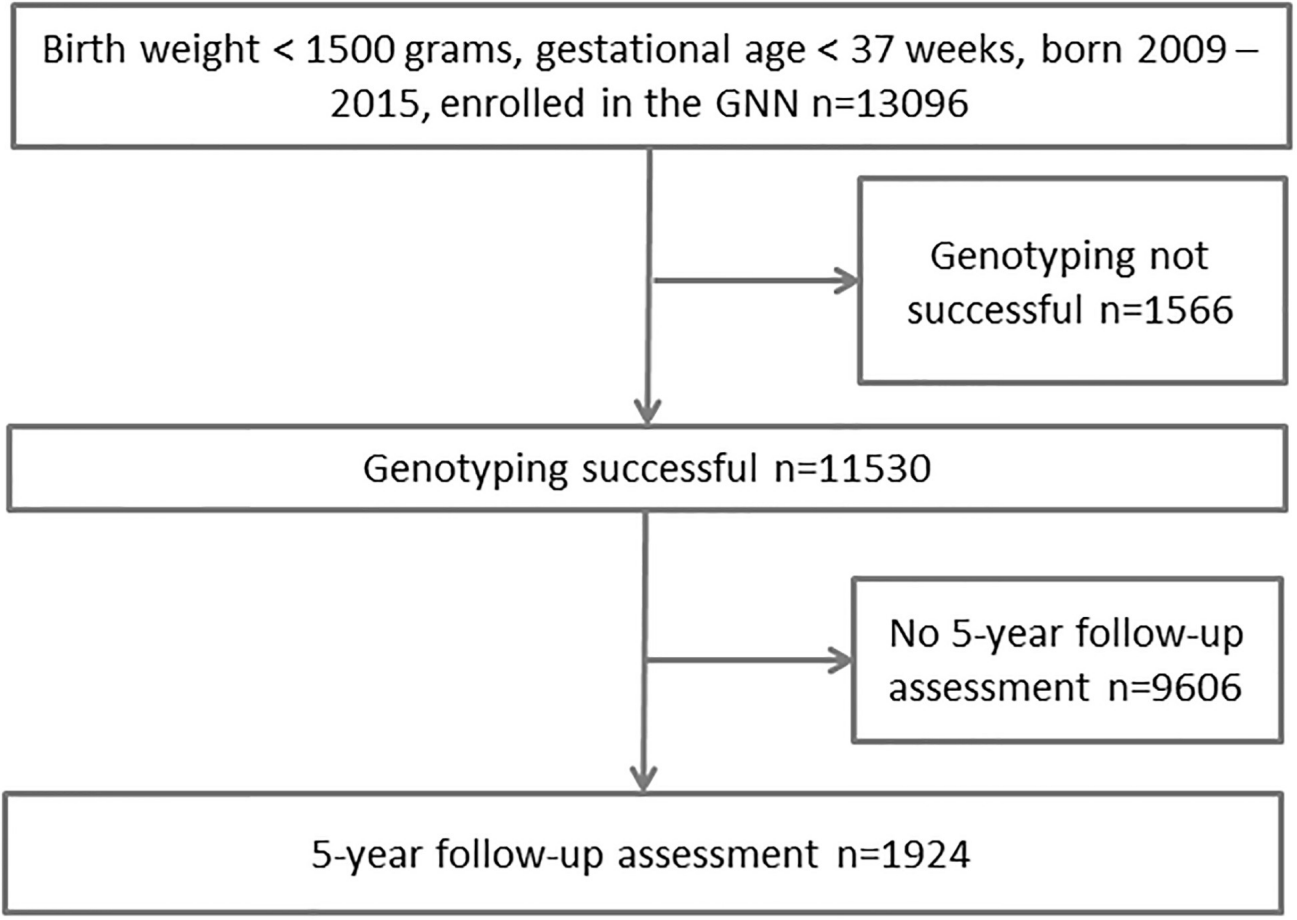
The study cohort.

Details of patient selection are given in Fig 1. Total number of eligible patients between 2009 and 2015 was 19136 (68% enrolment).

Baseline data of 1,924 preterm infants with 5-year follow up assessment are given in Table 1. Minor allele frequency was 0.44 for rs12794714, 0.30 for rs7944926 and 0.27 for rs2282679. All SNPs were in Hardy Weinberg equilibrium. To exclude the risk of “survival bias”, we analyzed mortality in all infants with successful genotyping. No significant differences were observed. Mortality in infants with low genetic score for vitamin D (n = 2,336) was 2.7% compared to 3.3% in infants with intermediate scores (n = 6,849) and 2.9% in infants with high genetic score for vitamin D (n = 2,345, p = 0.72 compared to low genetic score for vitamin D, Fisher’s exact test). Infants without 5-year follow up had a higher birth weight and gestational age, were less frequently multiples and more often SGA (S1 Table).

**Table 1:** Basic data of children with 5-year follow-up in relation genetic vitamin D level estimates.

Genetic score vitamin D levels	Low levels (< P20) (n = 387)	Intermediate levels(P20-P80) (n = 1,144)	High levels (> P80) (n = 393)	p*
Birth weight *[g]*	1,020 ± 291	1,003 ± 294	1,007 ± 301	0.538
Height at birth [cm]	36.1 ± 3,9	35.8 ± 3.8	35.7 ± 4.0	0.149
Head circumference at birth [cm]	25.5 ± 2,5	25.3 ± 2.6	25.3 ± 2.6	0.203
Weight at discharge [g]	2,610 ± 559	2,607 ± 591	2,655 ± 682	0.315
Height at discharge [cm]	46.3 ± 3,1	46.0 ± 3.5	46.1 ± 3.5	0.453
Head circumference at discharge [cm]	33.0 ± 2,2	32.9 ± 2.3	32.9 ± 2.5	0.449
Gestational age *[weeks]*	28.29 ± 2.4	28.11 ± 2.5	28.17 ± 2.5	0.497
Gender (Female) n *[%]*	187 (48.3)	546 (47.7)	204 (51.9)	0.352
Multiple birth n *[%]*	155 (40.1)	425 (37.2)	144 (36.6)	0.339
SGA infant n *[%]*	60 (15.5)	184 (16.1)	66 (16.8)	0.628

*p-values are indicated for low (< P20) vs. high levels (> P80) of vitamin D.

Birth weight and gestational age are indicated as mean ± standard deviation. Fisher’s exact test for gender, multiple birth and SGA; t-test for birth weight and gestational age. SGA = small-for-gestational-age.

Typical complications associated with a preterm birth are shown in Table 2. In this study, for very low birth weight infants with a lower genetic score for vitamin D no higher risk could be shown to develop one of the mentioned complications during their first stay in the hospital.

**Table 2:** Typical complications of preterm birth.

Genetic score vitamin D levels	Low levels (< P20) (n = 387)	Intermediate levels(P20-P80) (n = 1,144)	High levels (> P80) (n = 393)	p*
IVH n *[%]*	63 (16.3)	185 (16.2)	60 (15.3)	0.768
IVH III or IV *n [%]*	25 (6.5)	48 (4.2)	18 (4.6)	0.275
PVL n *[%]*	5 (1.3)	26 (2.3)	8 (2.0)	0.578
BPD n *[%]*	55 (15.0)	209 (18.3)	73 (18.6)	0.213
Sepsis n *[%]*	57 (14.7)	168 (14.7)	69 (17.6)	0.287
NEC with surgery n *[%]*	7 (1.8)	24 (2.1)	10 (2.5)	0.625
ROP *n [%]*	11 (2.8)	45 (3.9)	14 (3.6)	0.685

*p-values are indicated for low (< P20) vs. high levels (> P80) of vitamin D.

All p-values are derved from Fisher’s exact test. IVH = intraventricular hemorrhage, PVL = periventricular leukomalacia, BPD = bronchopulmonary dysplasia, NEC = necrotizing enterocolitis, ROP = retinopathy of prematurity.

Growth data (height, weight, body mass index and head circumference) at the age of five did not differ with regard to gsvitD status (Table 3).

**Table 3:** Body measurements at the follow-up examination after five years.

Genetic score vitamin D levels	Low level(< P20) (n = 387)	Intermediate levels(P20-P80) (n = 1,144)	High levels(> P80) (n = 393)	p*
Weight at 5 years *[kg]*	18.70 ± 3.4	18.57 ± 3.4	18.37 ± 3.1	0.164
Head circumference at 5 years *[cm]*	50.32 ± 1.7	50.30 ± 1.9	50.28 ± 1.8	0.782
Height at 5 years *[cm]*	112.74 ± 6.2	112.37 ± 6.2	111.83 ± 5.9	0.036
*BMI [kg{m*^*2*^*]*	14.6 ± 1.5	14.6 ± 1.6	14.6 ± 1.7	0.861

*p-values are indicated for low (< P20) vs. high levels (> P80) of vitamin D.

All values are indicated as mean ± standard deviation; p-values are derived from t-test.

Children with low gsvitD were significantly taller (one centimeter) than children with high gsvitD. This result does not remain significant after being adjusted by the Bonferroni correction (adjusted p-value for 9 comparisons = 0.324).

**Fig 2:**
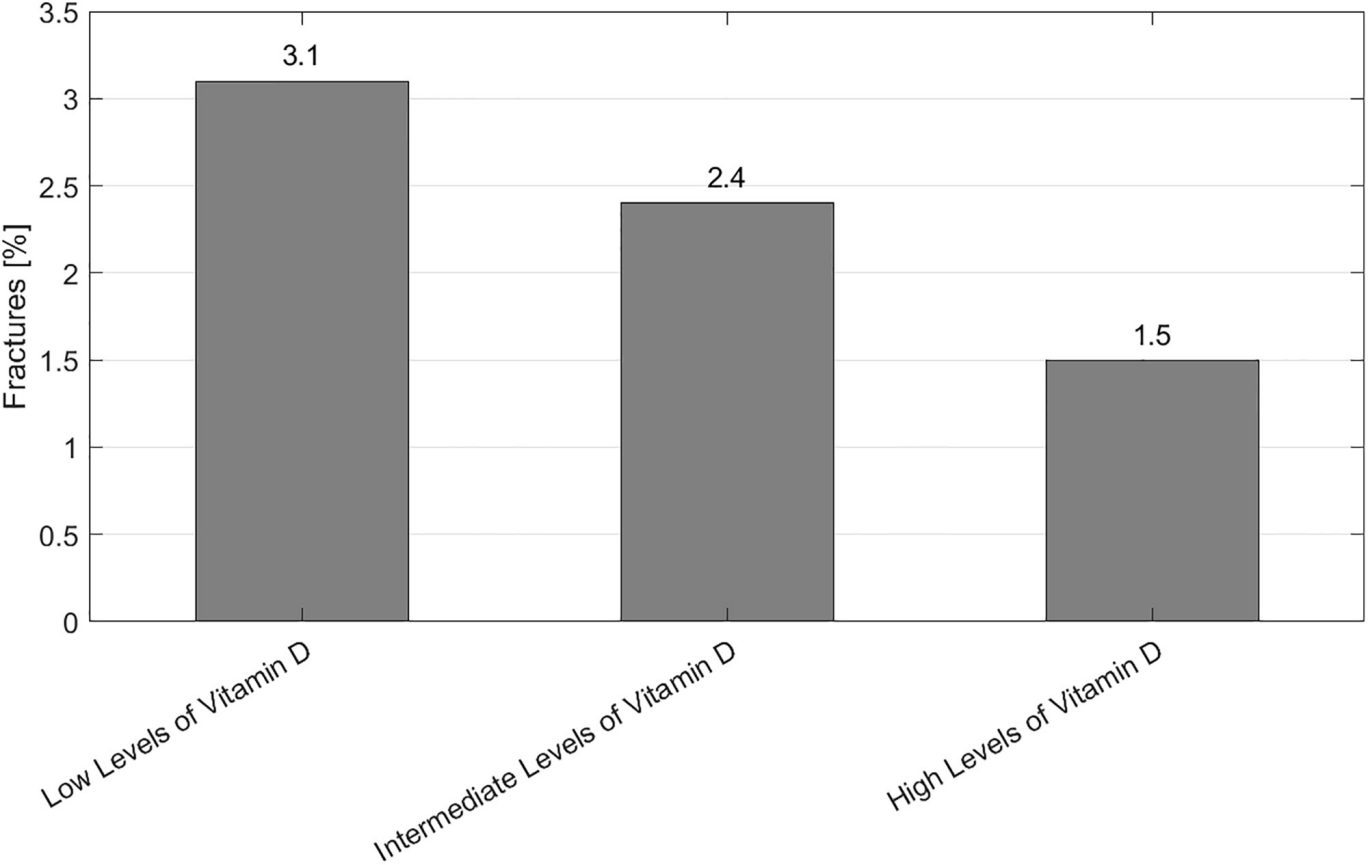
Percental occurrence of bone fractures of the examined children until the age of five.

Fig 2 shows frequencies of bone fractures stratified for gsvitD. This figure implies that children with higher gsvitD had fewer fractures but differences between infants with high and low gsvitD did not reach statistical significance (p = 0.159, Fisher’s exact test, adjusted p = 1.0). Since prevalence of fractures at five years is very low, follow up of about 7,700 genotyped infants would be needed to confirm a risk difference of 1.5% (1.5% fracture risk in preterm infants with high genetic score vitamin D levels vs. 3.0% fracture risk in preterm infants with low genetic score vitamin D levels, for type I error level < 0.05 and power > 0.8).

## Discussion

None of the aforementioned associations of a vitamin D deficiency could be confirmed by this work.

A number of publications report an association between a decreased vitamin D level in the mother or the child with relevant short- and long-term endpoints [2–8]. Most of these studies measured vitamin D levels in the blood. Since approximately three quarters of the variability of the vitamin D level can be explained by genetic factors [14], more recent publications already use the calculation of the vitamin D levels based on individual genetic variables [6,21]. For this reason, we used a genetically estimated vitamin D level for this study.

A probably more precise record of the vitamin D level would have been the measurement of the 25-hydroxyvitamin D. This would have required a blood sample of the children which could not have been performed in such a high number of participants.

Leffelaar et al. and Bodnar et al. both showed an association between a vitamin D deficiency of the mother (measurement of 25(OH)D in the early pregnancy) and a higher occurrence of SGA infants [22,23]. Due to these studies, we pursued the hypothesis, that infants with a genetic predisposition for vitamin D deficiency would be more frequently SGA infants. As an extension we tested this hypothesis according to birth weight, gestational age and weight, height and head circumference at five years. In this study, all data collected are remarkably similar and without differences between infants with low, intermediate and high genetic vitamin D level estimates. Our results are in line with the findings of a recently published study by Roth et al., showing no impact of a vitamin D supplementation of healthy pregnant women on the longitudinal growth of their babies at the age of one [24].

In adults, vitamin D deficiency is associated with increased risk for neurological complications, such as cerebral microangiopathies, lacunes and severe white matter hyperintensity [8]. We tested preterm infants with high and low vitamin D level estimates for neurologic complications of prematurity like IVH and PVL but were not able to detect any differences. This was also true for other complications of prematurity.

In the first year of life, preterm birth and vitamin D deficiency is associated with an excess risk of fractures [25]. It is notable, that we saw a not statistically significant trend to less fractures in children with high genetic vitamin D level estimates. However, only 45 of 1924 (2.3%) children had a bone fractures until the age of five. It has to be investigated in even larger sample sizes if the small difference observed in our cohort is actually associated with insufficient vitamin D levels and if additional supplementation of vitamin D to these infants can be helpful.

The findings of this study support two large recently published randomized studies by Roth et al. and Manson et al. Latter could not show any positive effects of a vitamin D supplementation on the growth of infants [24] or on a lower incidence of cancer or cardiovascular complications [26].

Our study has several limitations. We were not able to measure plasma vitamin D levels, which might be more reliable than genetic estimates since the effect of genetics might be diluted by dietary intake and vitamin D supplementation. However, plasma levels can alter from day to day and could be less informative for lifelong vitamin D status than genetic estimates. Furthermore, we did not assess the number of children with vitamin D supplementation up to the age of 5 years. Since prolonged use of vitamin D supplements might be more common in premature infants and is associated with reduced fracture risk [27] our data are not representative for healthy neonates.

Furthermore, the selection of the examined SNPs influences the data. Jiang et al. were able to replicate four known and two new genome-wide significant genetic loci influencing 25-hydroxyvitamin D concentration [28]. Three of these loci are congruent to the ones used for this study. However, we acknowledge that our findings might differ if a variant genetic method was applied (different SNPs and SNP genotyping) and if the case number had been even larger. Finally, standardized radiologic assessment of bone structure during the stay in the hospital is not part of the GNN-protocol. Therefore, we were not able to test the association between genetically estimated vitamin D levels and rickets of prematurity.

Strengths of our study include the large sample size and the highly standardized long-term follow-up. However, for rare endpoints like bone fractures even our sample size was not sufficient.

We analyzed genetic factors altering vitamin D levels in a large cohort of preterm infants to determine if infants with a genetic predisposition for low vitamin D levels are at higher risk for short term complications of prematurity or long term growth failure and bone fractures. Despite the large study population our data shows no association between low genetic vitamin D level estimates and adverse outcome of very low birth weight infants.

## Supporting information

S1 Table Missing analysis. Data for genotyped infants with 5-year follow-up are given in Tables 1 and 2. P-values are indicated for genotyped children with or without 5-year follow up data. Birth weight and gestational age are indicated as mean ± standard deviation. t-test for birth weight and gestational age, Fisher’s exact test for the remaining data. SGA = small-for-gestational-age, IVH = intraventricular hemorrhage, PVL = periventricular leukomalacia, BPD = bronchopulmonary dysplasia, NEC = necrotizing enterocolitis.
(TIFF)
