# Supplementary figures and images for: Genetic predisposition for vitamin D deficiency is not associated with adverse outcome of very low birth weight infants: A cohort study from the German Neonatal Network
Source: PLoS One. 2020 Mar 31;15(3):e0230426. doi: 10.1371/journal.pone.0230426 (PMC7108707; doi:10.1371/journal.pone.0230426)

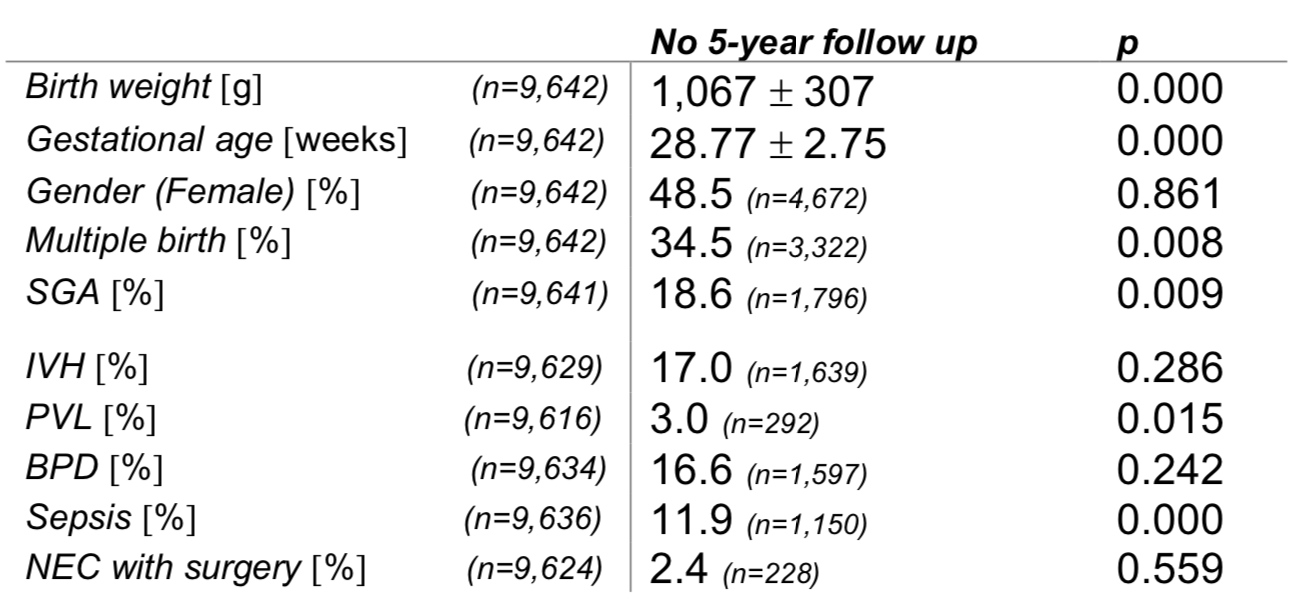

Supplement: S1 Table — Data for genotyped infants with 5-year follow-up are given in Tables 1 and 2. P-values are indicated for genotyped children with or without 5-year follow up data. Birth weight and gestational age are indicated as mean ± standard deviation. t-test for birth weight and gestational age, Fisher’s exact test for the remaining data. SGA = small-for-gestational-age, IVH = intraventricular hemorrhage, PVL = periventricular leukomalacia, BPD = bronchopulmonary dysplasia, NEC = necrotizing enterocolitis. (TIFF) [file pone.0230426.s001.tiff]
